# Supplementary material for: APE1 active site residue Asn174 stabilizes the AP site and is essential for catalysis
Source: J Biol Chem. 2025 Sep 1;301(10):110655. doi: 10.1016/j.jbc.2025.110655 (PMC12557600; doi:10.1016/j.jbc.2025.110655)
Supplement: Supplemental Figures [file mmc1.docx]

**SUPPORTING INFORMATION**

APE1 active site residue Asn174 stabilizes the AP site and is essential for catalysis

Kaitlin M. DeHart, Nicole M. Hoitsma, Spencer H. Thompson, Veniamin A. Borin, Pratul K. Agarwal, and Bret D. Freudenthal

Supporting Information Includes:

Supplemental Figures S1-S6


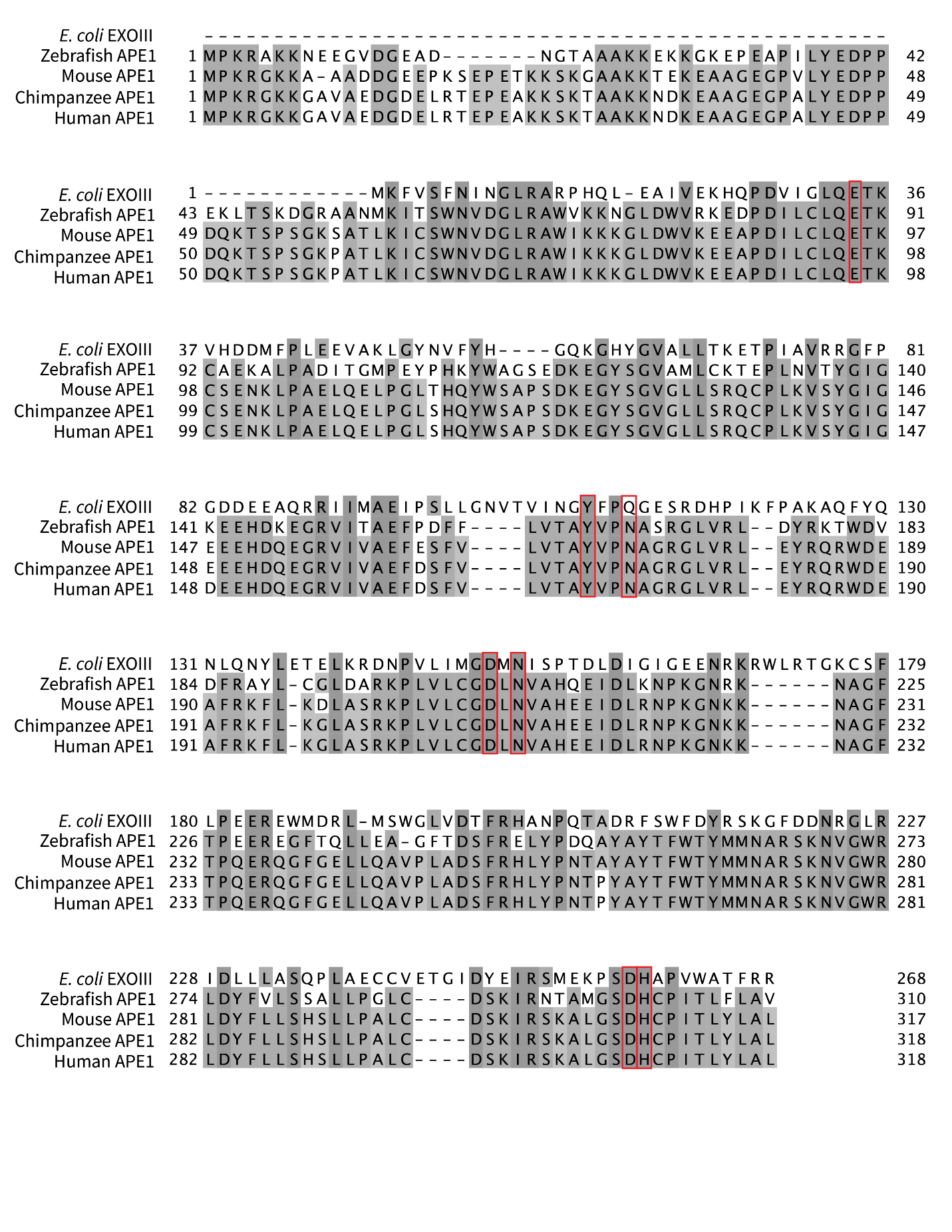


Supplemental Figure S1: **Multiple sequence alignment of primary AP-endonucleases across five selected species**

Full amino acid sequences for human APE1 (Uniprot ID: P27695), chimpanzee APE1 (Uniprot ID: A2T6Y4), mouse APE1 (Uniprot ID: P28352), zebrafish APE1 (Uniprot ID: A0MTA1), and E. coli Exonuclease III (Uniprot ID: P09030) were aligned using ClustalOmega. The multiple sequence alignment was visualized using JalView v 2.11.4.1 and colored by consensus sequence where sequence identity was greater than or equal to 60% across the five species. Red boxes outline active site residues.


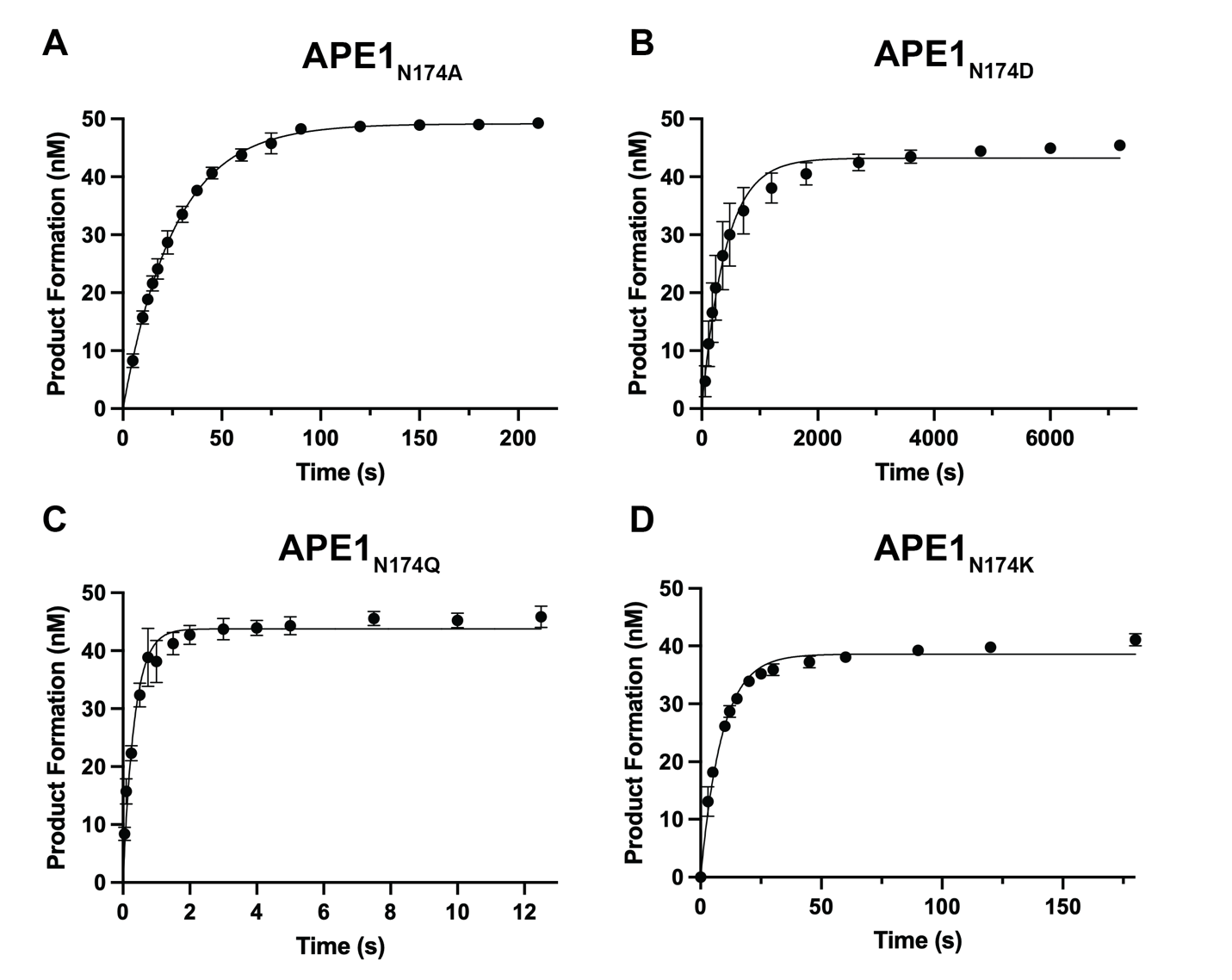


Supplemental Figure S2: **Fits of single turnover analysis of APE1 N174 mutants**

The amount of product formation over time under single turnover conditions was analyzed to determine the cleavage rate constant, *k_obs_*, for APE1 N174 mutants (N=3-4). Error bars are smaller than can be displayed when not present. The fits of all replicates from single turnover kinetic analysis for (A) the APE1_N174A_ mutant, (B) the APE1_N174D_ mutant, (C) the APE1_N174Q_ mutant, and (D) the APE1_N174K_ mutant are shown above.


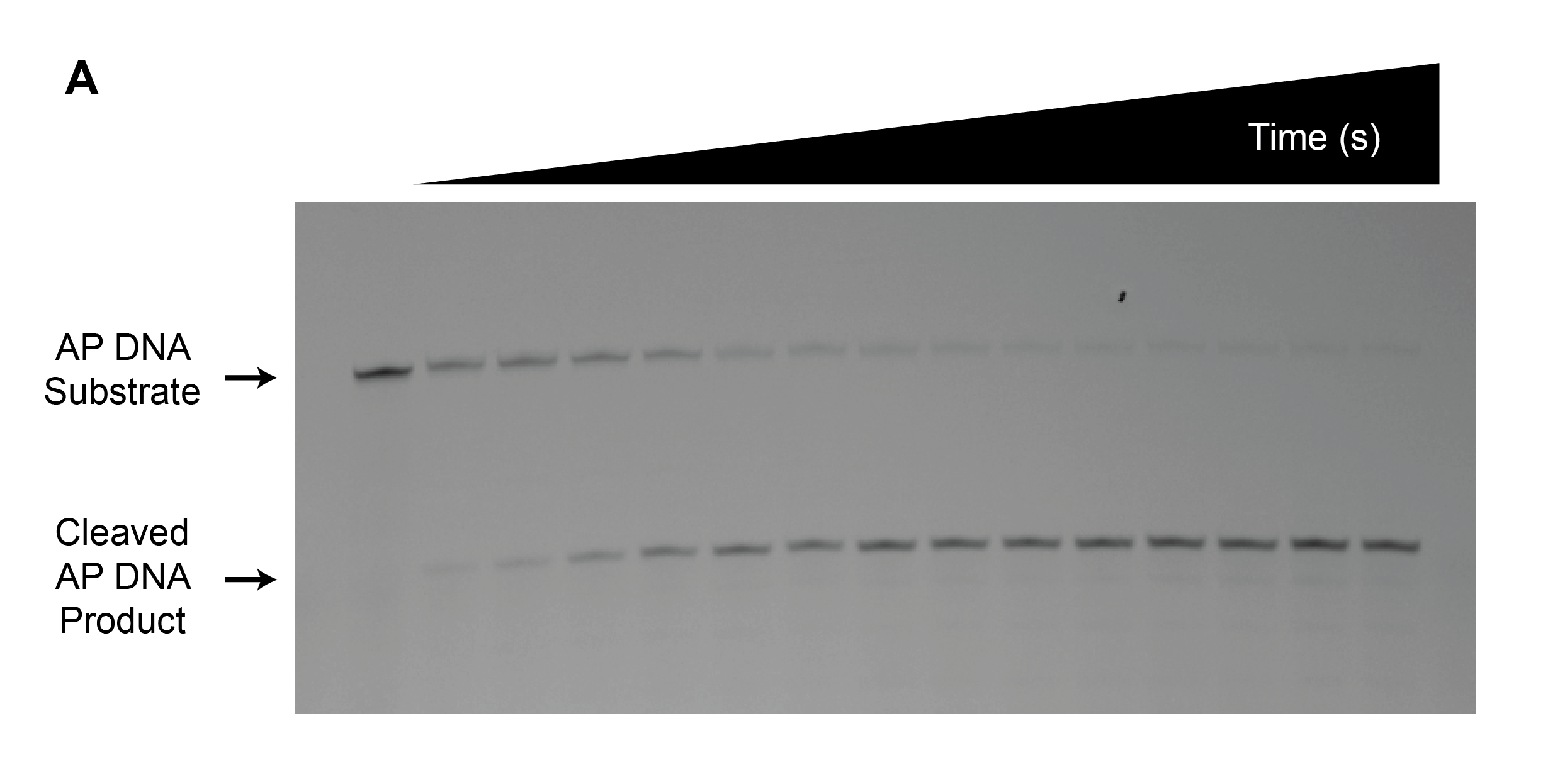


Supplemental Figure S3: **Exemplary Gel of Single Turnover Kinetics Experiments**

A UreaGel was used to visualize the amount of product formed by each of our APE1 mutants over time under single turnover conditions. Here we have provided a representative gel from a single-turnover kinetic experiment of the APE1_N174Q_ mutant cleaving THF AP DNA.


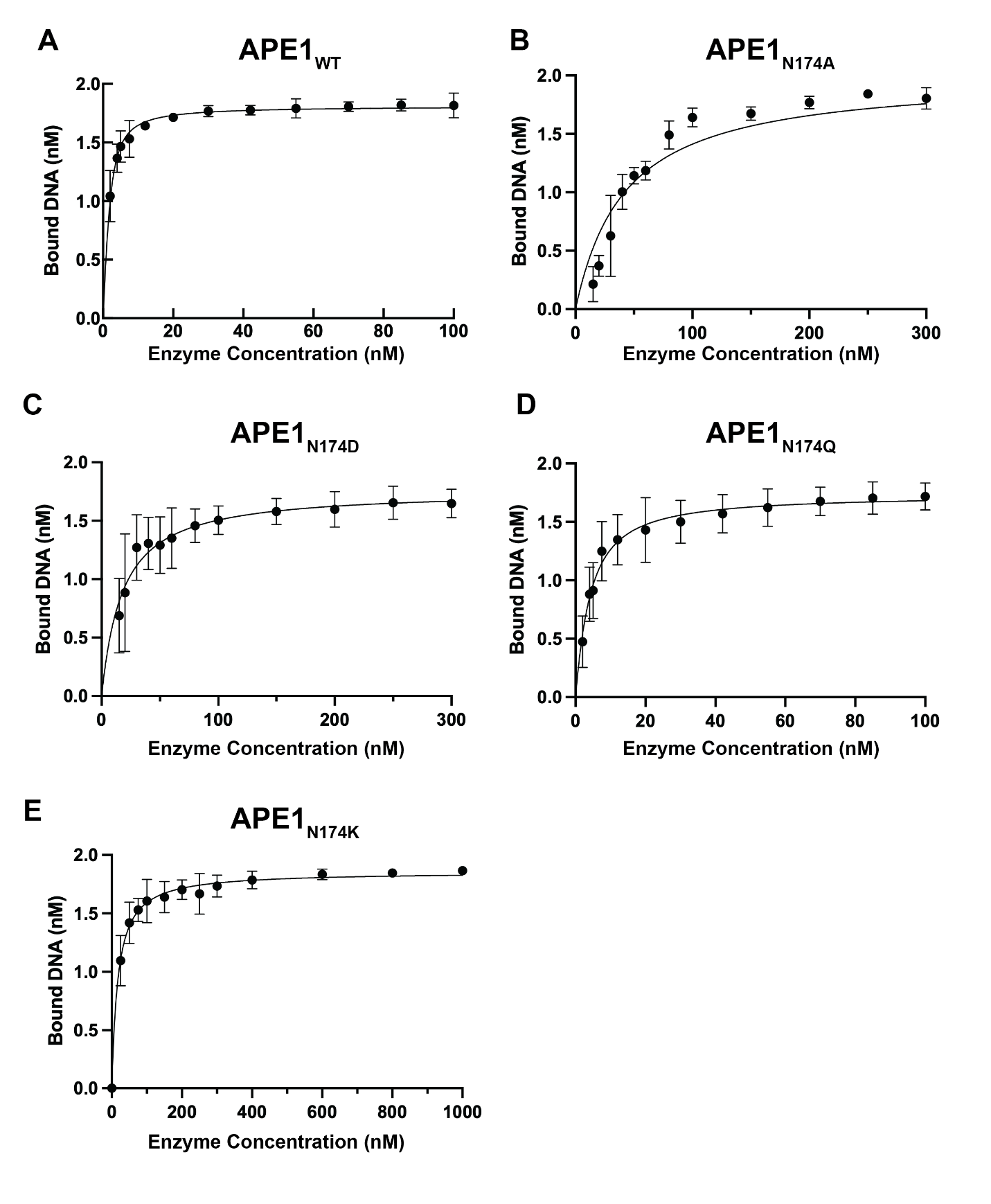


Supplemental Figure S4: **Fits of electrophoretic mobility shift assays (EMSAs) of APE1 N174 mutants**

The amount of DNA bound by APE1 mutants vs the concentration of mutant APE1 was analyzed to determine the apparent binding affinity, *K*_D App_, for APE1 and all APE1 N174 mutants (N=3-5). Error bars are smaller than can be displayed when not present. The fits of all replicates from EMSAs for (A) APE1_WT_, (B) the APE1_N174A_ mutant, (C) the APE1_N174D_ mutant, (D) the APE1_N174Q_, and (E) the APE1_N174K_ mutant are shown above.


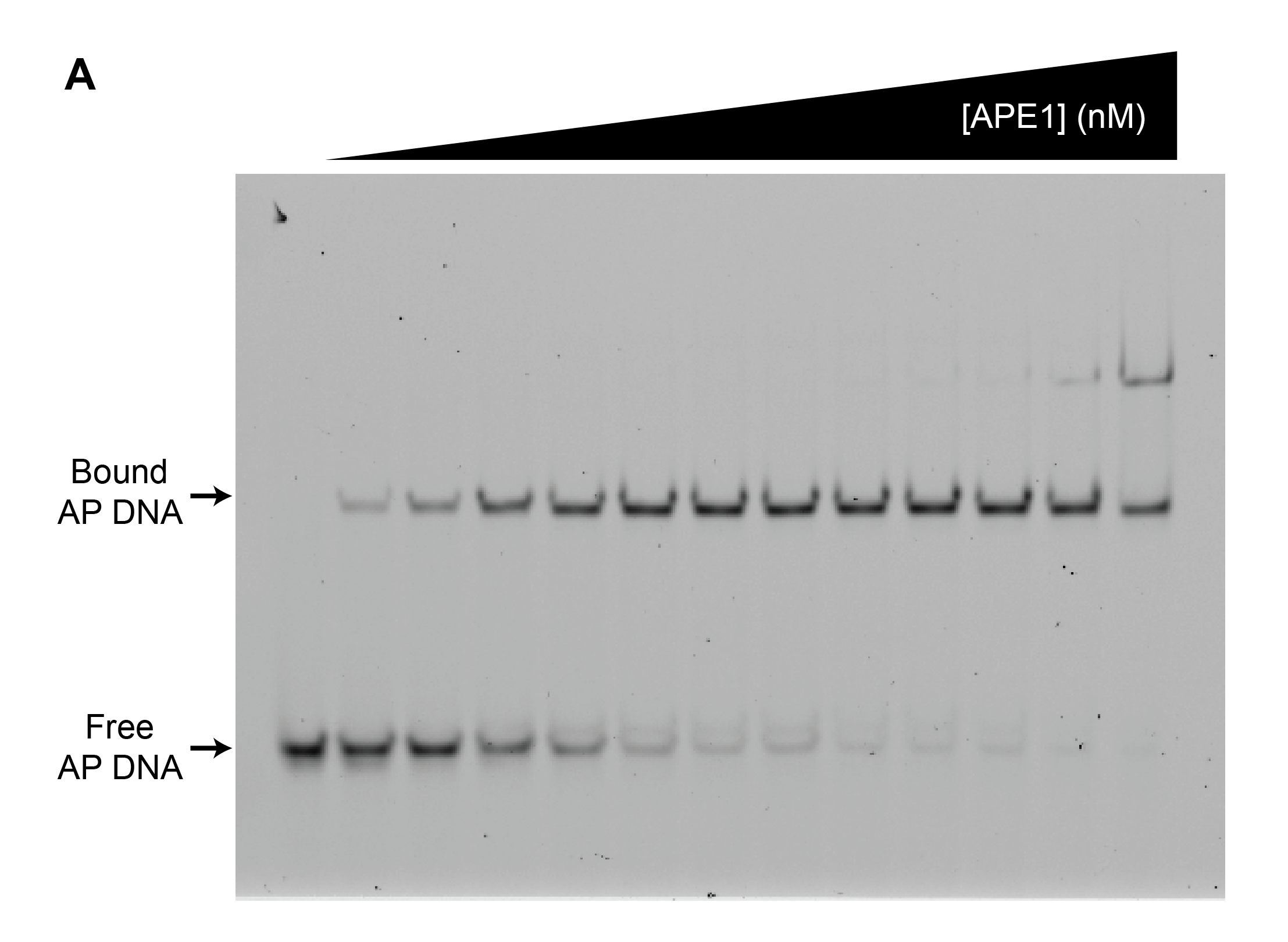


Supplemental Figure S5: **Exemplary EMSA Gel**

A native gel was used to visualize the fraction of free AP DNA and the fraction of AP DNA bound by increasing concentrations of APE_WT_ and each of our APE1 mutants. Here we have provided a representative gel from an EMSA experiment of APE1_WT_ binding to THF AP DNA.


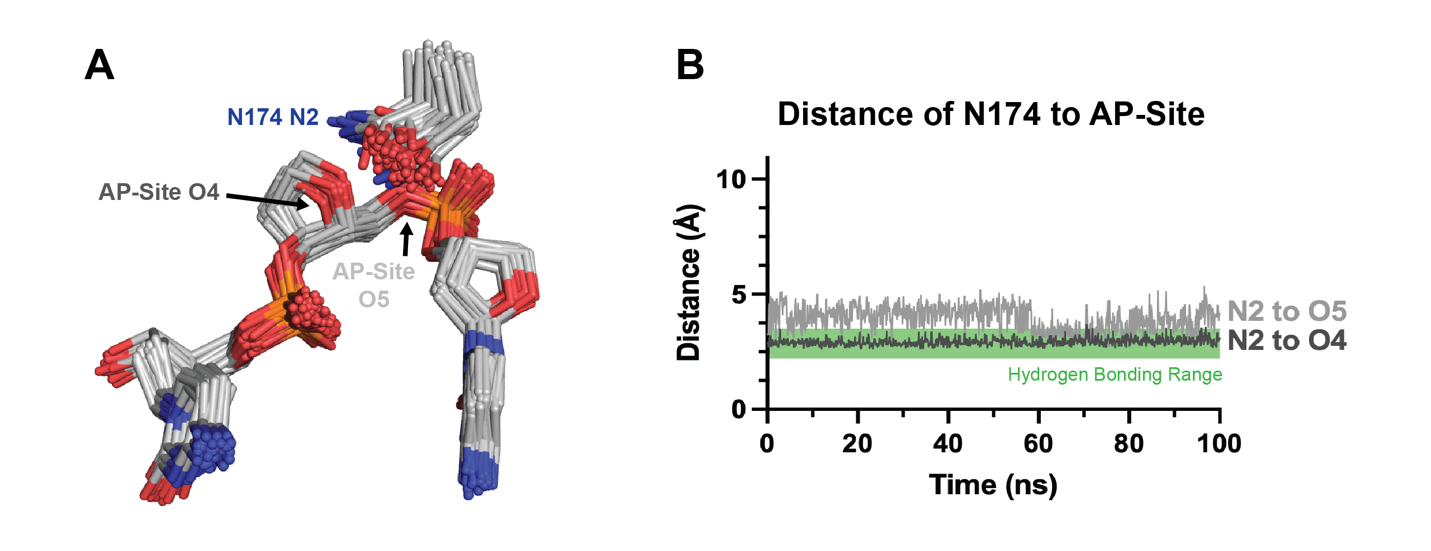


Supplemental Figure S6: **Analysis of APE1_WT_ rotamer conformations and hydrogen bonding distances from computational simulations**

Computational modeling was performed to determine rotamer states of residue 174 and the resulting distances to the AP site in the APE1_WT_ substrate complex. (A) Rotamer states of N174 in the APE1_WT_ substrate complex were determined by computational modeling. Rotamers clustered near the standard rotamer states m-20 and m-80 (both Χ_1_=-71). (B) The distances between N2 of N174 and O4 and O5 of the AP site were plotted to determine if N174 is within hydrogen bonding distance of the AP site in the APE1_WT_ substrate complex. Hydrogen bonding range is marked by the green box on distance plots as distances between 2.2 and 3.5 Å. Here, N2 of N174 in the APE1_WT_ substrate complex enters hydrogen bonding distance of either O4 or O5 of the AP site in our computational simulations.
